# Supplementary material for: StarBEAST2 Brings Faster Species Tree Inference and Accurate Estimates of Substitution Rates
Source: Mol Biol Evol. 2017 Apr 14;34(8):2101–14. doi: 10.1093/molbev/msx126 (PMC5850801; doi:10.1093/molbev/msx126)
Supplement: Supplementary Data [file msx126_supp.pdf]

Supplementary material for StarBEAST2

Huw A. Ogilvie*[1,2], Remco R. Bouckaert[2,3], and Alexei J. Drummond[2,3]

[1]Division of Ecology and Evolution, Research School of Biology, Australian National University, Canberra, Australia

[2]Centre for Computational Evolution, University of Auckland, Auckland, New Zealand

[3]Department of Computer Science, University of Auckland, Auckland, New Zealand

March 31, 2017

*huw.ogilvie@anu.edu.au

1 Supplementary figures

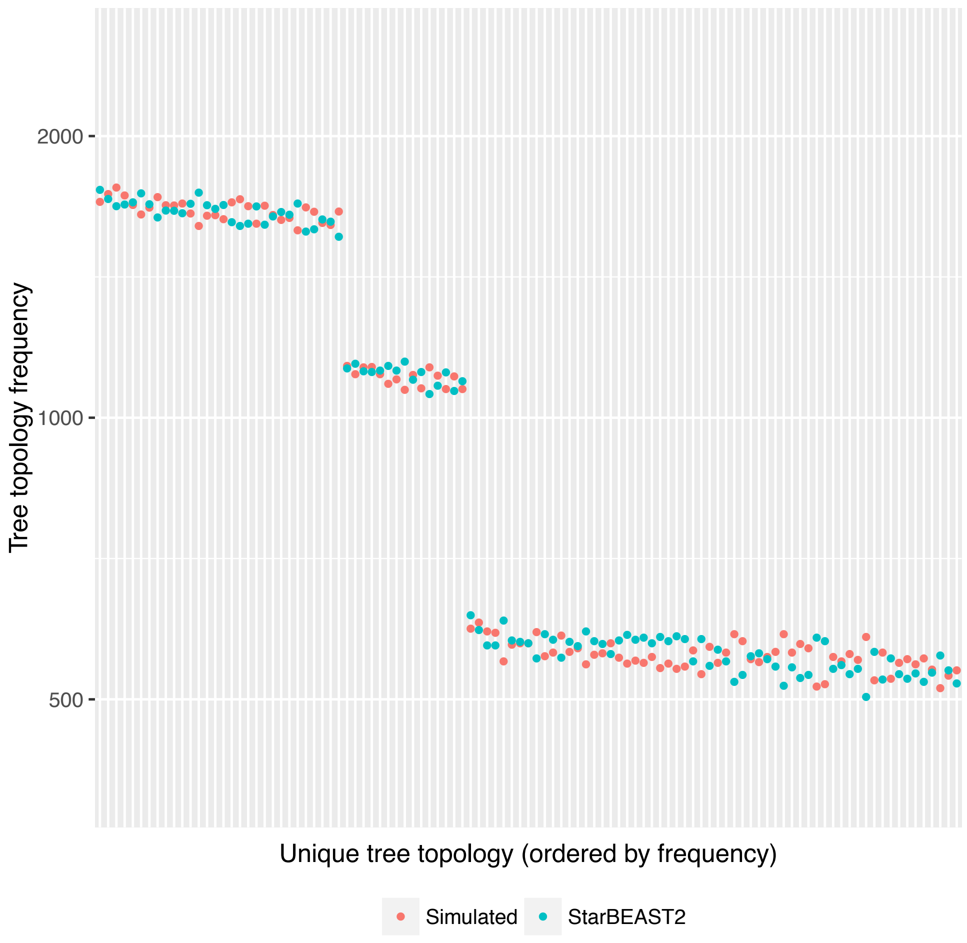

Figure S1: Frequency of five-taxon species tree topologies sampled from a birth-death prior distribution. Topologies were sampled by simulating trees using biopy (red), or by using a StarBEAST2 MCMC chain (blue). Frequencies are identical apart from noise, indicating that StarBEAST2 is mathematically correct. Three levels of probability are evident from left to right — high probability balanced topologies e.g. ((a,b),((c,d),e)), middle probability intermediate topologies e.g. (((a,b),(c,d)),e), and low probability unbalanced topologies e.g. ((((a,b),c),d),e).

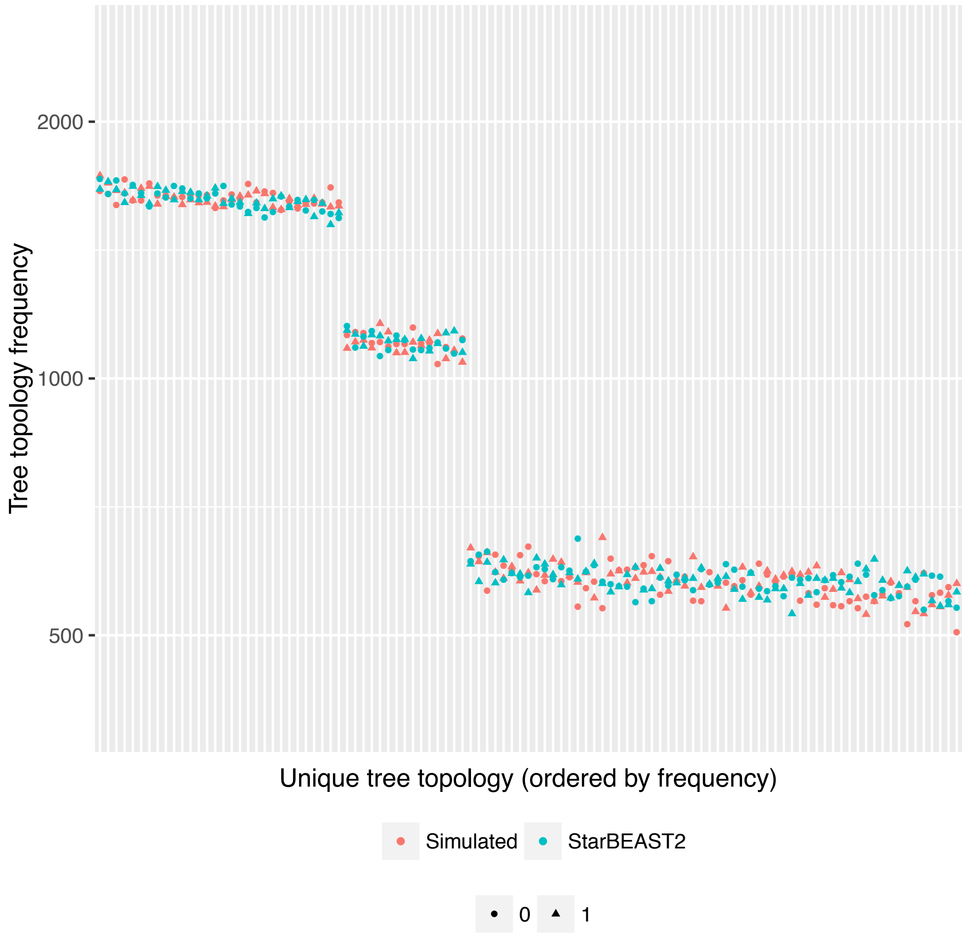

Figure S2: Frequency of five-taxon gene tree topologies sampled from a multispecies coalescent prior distribution. Topologies were sampled by simulating gene trees within species trees using biopy (red), or by using a StarBEAST2 MCMC chain (blue). Gene trees were sampled with two clock rates, 0.5 (circles) and 2.0 (triangles), although clock rate should not affect topology or node heights in units of time. Frequencies are identical apart from noise, indicating that StarBEAST2 is mathematically correct. Three levels of probability are evident from left to right — high probability balanced topologies e.g. ((a,b),((c,d),e)), middle probability intermediate topologies e.g. (((a,b),(c,d)),e), and low probability unbalanced topologies e.g. ((((a,b),c),d),e).

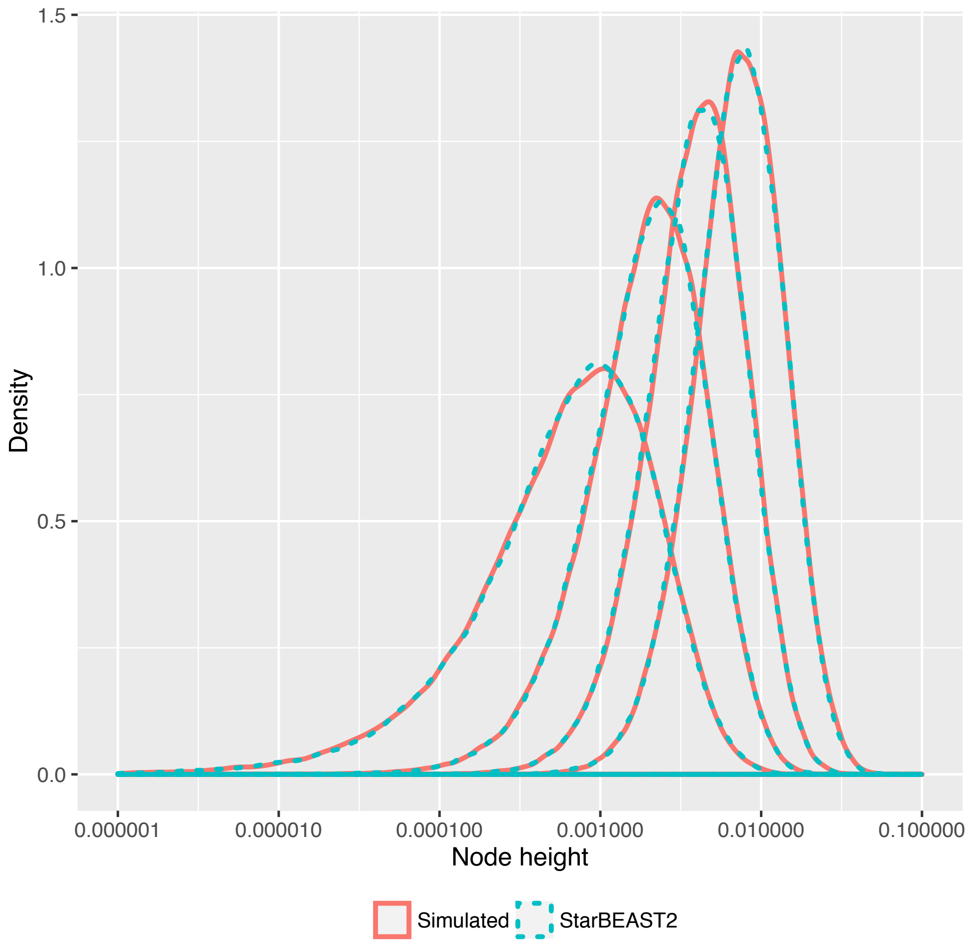

Figure S3: Probability densities of five-taxon species tree node heights sampled from a birth-death prior distribution. Node heights were sampled by simulating trees using biopy (red), or by using a StarBEAST2 MCMC chain (blue). Probability densities are plotted separately for each ranked node giving four peaks, one for each internal node. Node height probability densities are identical apart from noise, indicating that StarBEAST2 is mathematically correct.

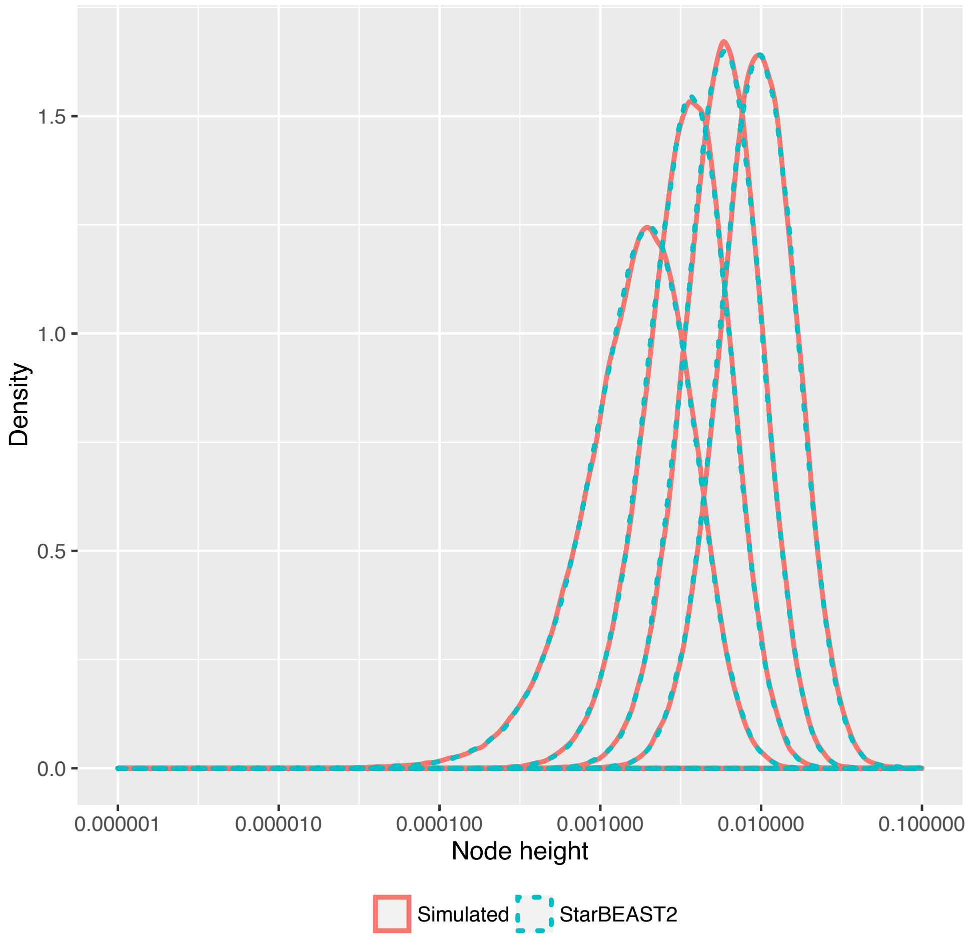

Figure S4: Probability densities of five-taxon gene tree node heights sampled from a multispecies coalescent prior distribution. Node heights were sampled by simulating gene trees within species trees using biopy (red), or by using a StarBEAST2 MCMC chain (blue). Gene trees were sampled with two clock rates, 0.5 and 2.0, and node heights from both sets of gene trees were combined as clock rate should not affect topology or node heights in units of time. Probability densities are plotted separately for each ranked node giving four peaks, one for each internal node. Node height probability densities are identical apart from noise, indicating that StarBEAST2 is mathematically correct.

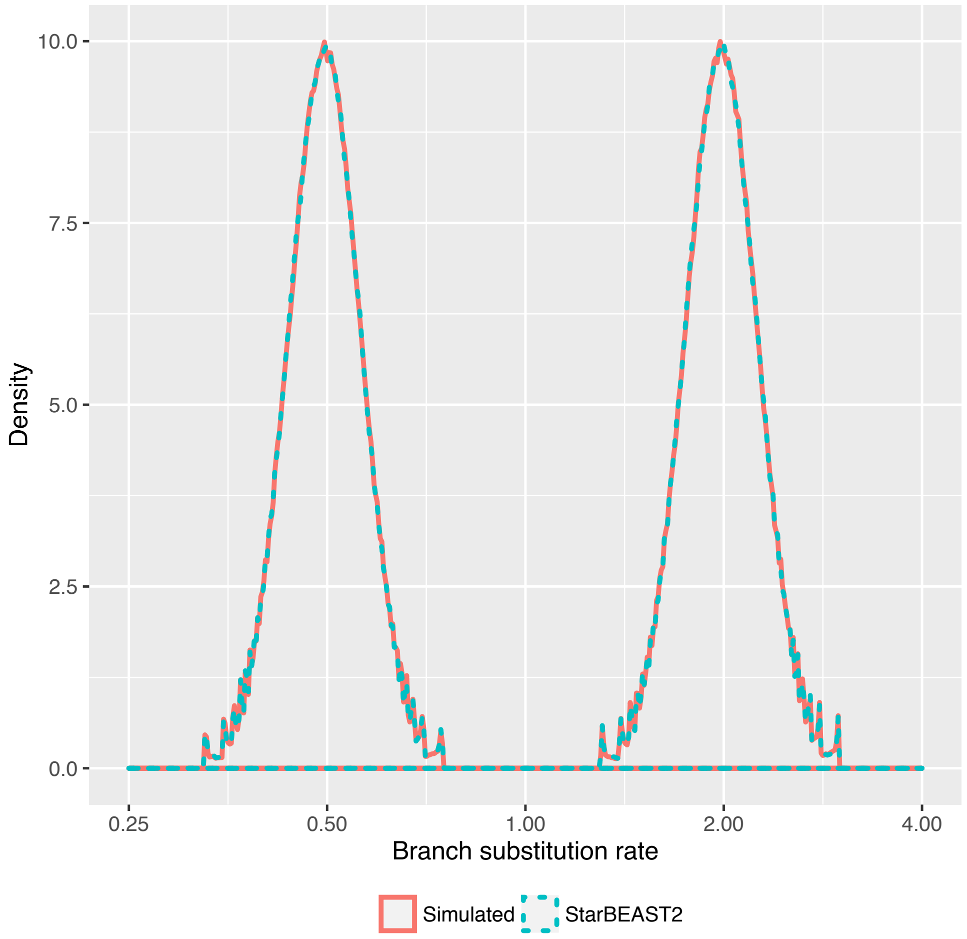

Figure S5: Probability densities of five-taxon gene tree branch rates produced by a species tree uncorrelated lognormal (UCLN) relaxed clock. Branch rates were sampled by simulating gene trees within species trees using biopy and simulating species tree branch rates using scipy (red), or by using a StarBEAST2 MCMC chain (blue). Gene trees were sampled with two clock rates, 0.5 and 2.0, resulting in two peaks. Branch rate probability densities are identical apart from noise, indicating that StarBEAST2 is mathematically correct.

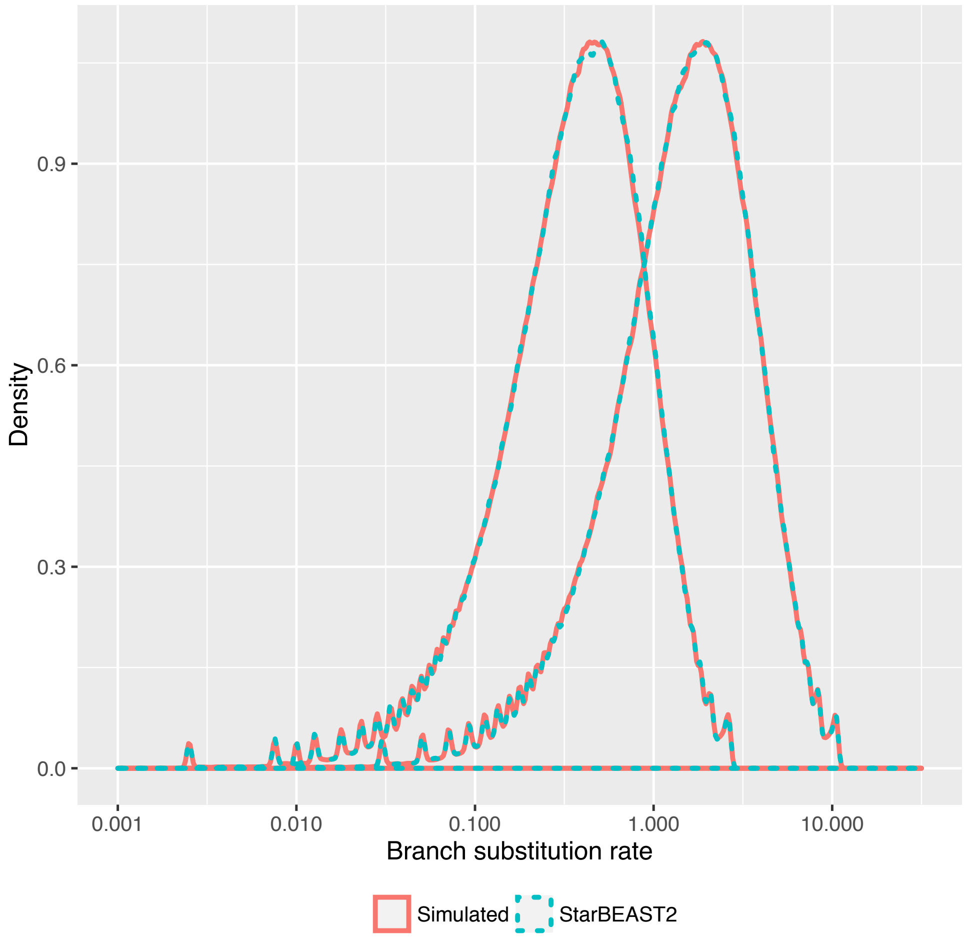

Figure S6: Probability densities of five-taxon gene tree branch rates produced by a species tree uncorrelated exponential (UCED) relaxed clock. Branch rates were sampled by simulating gene trees within species trees using biopy and simulating species tree branch rates using scipy (red), or by using a StarBEAST2 MCMC chain (blue). Gene trees were sampled with two clock rates, 0.5 and 2.0, resulting in two peaks. Branch rate probability densities are identical apart from noise, indicating that StarBEAST2 is mathematically correct.

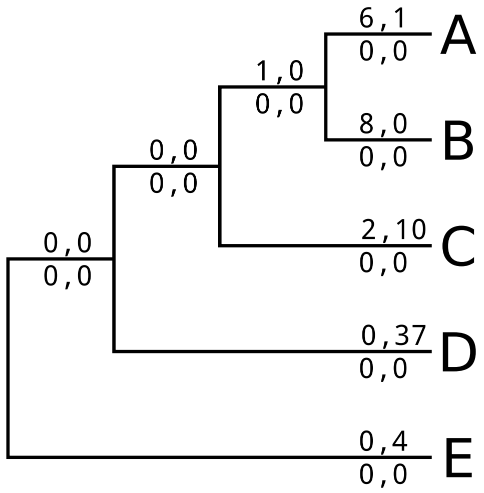

Figure S7: Number of erroneous per-species substitution rates estimated using BEAST concatenation and StarBEAST2. Erroneous branch rates are those with 95% highest posterior density (HPD) credible intervals which do not include the true rate of 1. Numbers above a branch are the counts of erroneous branches for concatenation, and those below are the counts for StarBEAST2, both out of 96 replicates. The first count is the number of branch rates inferred to be faster than 1, and the second is the number inferred to be slower than 1.

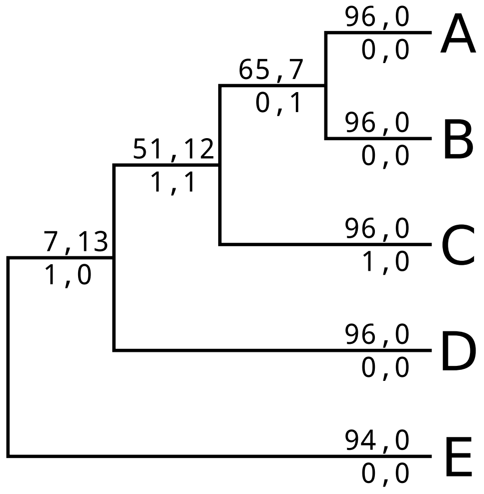

Figure S8: Number of erroneous branch lengths estimated using BEAST concatenation and StarBEAST2. Erroneous branch lengths are those with 95% highest posterior density (HPD) credible intervals which do not include the true simulated length for a given branch. Numbers above a branch are the counts of erroneous branches for concatenation, and those below are the counts for StarBEAST2, both out of 96 replicates. The first count is the number of branch lengths inferred to be longer than the true length, and the second is the number inferred to be shorter.

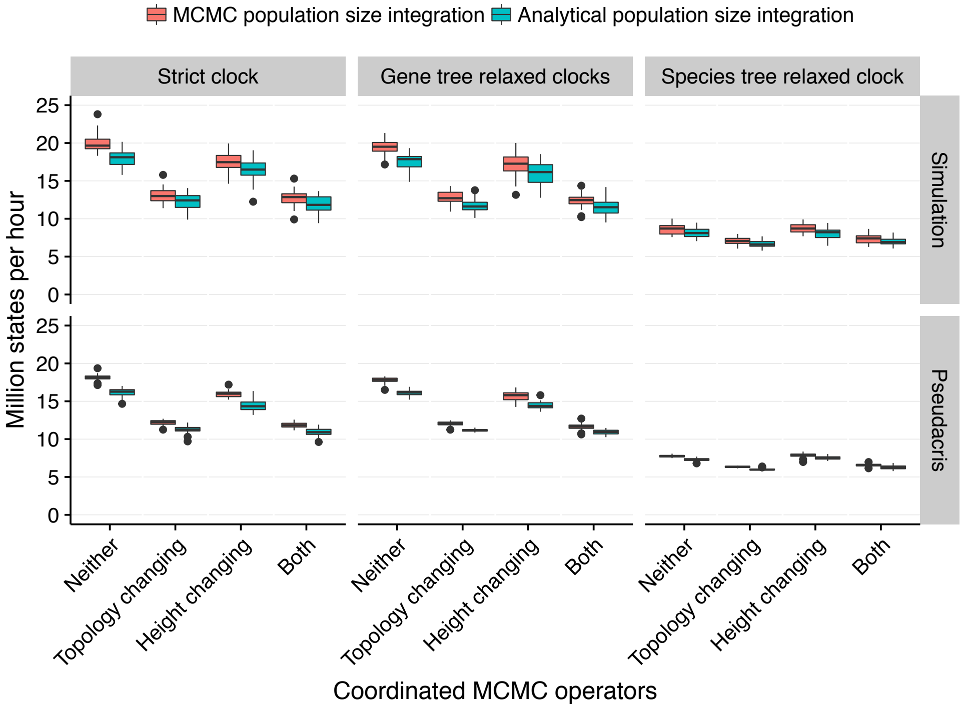

Figure S9: Impact of operators, population size integration and clock models on the calculation time for each state. Topology refers to the replacement of naïve nearest-neighbor interchange and subtree prune and regraft operators with coordinated operators. Height refers to the addition of operators which make coordinated changes to node heights. Uncorrelated log-normal relaxed clocks were applied to either each gene tree (GT-UCLN) or to the species tree (ST-UCLN). N = 30.

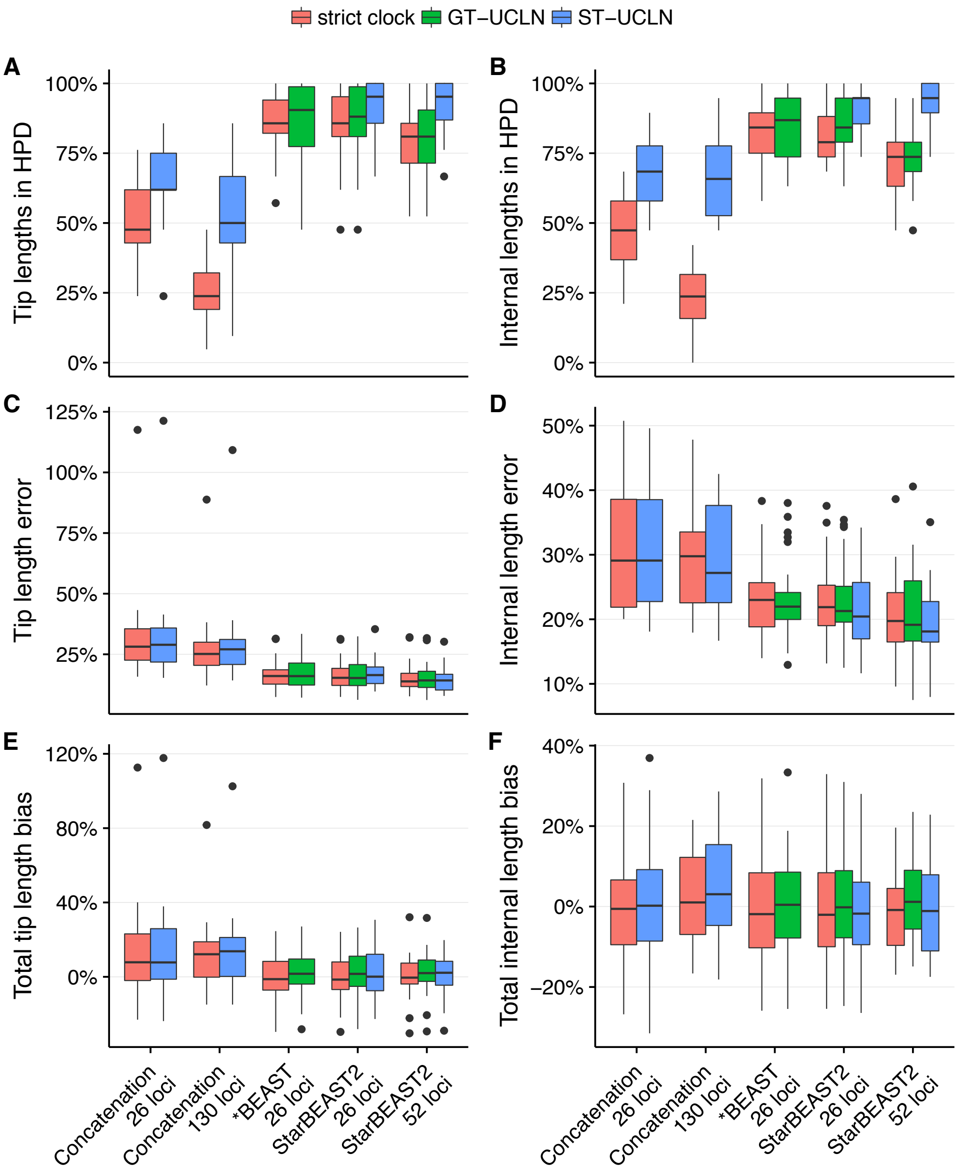

Figure S10: Coverage and accuracy of species branch lengths using different methods. Methods are StarBEAST2, *BEAST and unphased BEAST concatenation with uncorrelated log-normal relaxed clocks applied to each gene tree (GT-UCLN) or to the species tree (ST-UCLN). (A,B) The percentages of true branch lengths present within the corresponding 95% highest posterior density (HPD) credible intervals. (C,D) The difference between the sum of estimated branch lengths and the sum of true branch lengths as a percentage of the sum of true branch lengths. (E,F) The sum of absolute differences between estimated and simulated branch lengths as a percentage of true tree length. N = 30.

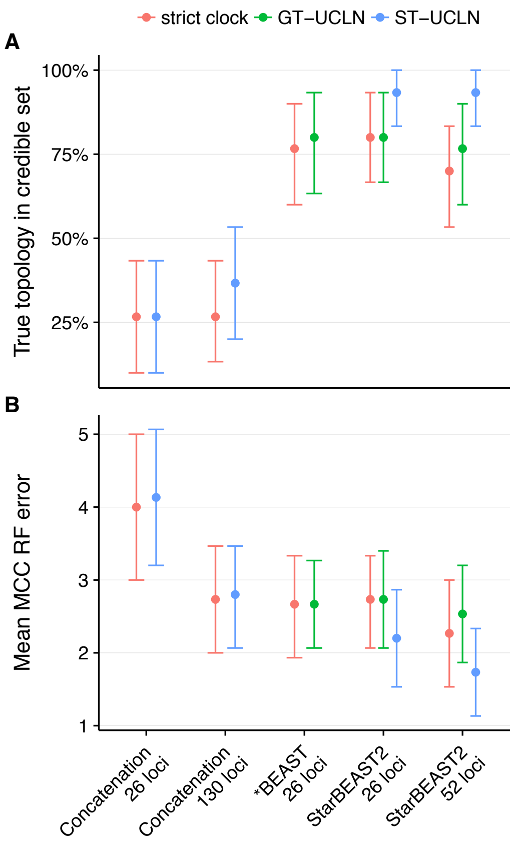

Figure S11: Coverage and accuracy of species tree topologies using different methods. Methods are StarBEAST2, *BEAST and unphased BEAST concatenation with uncorrelated log-normal relaxed clocks applied to each gene tree (GT-UCLN) or to the species tree (ST-UCLN). (A) The percentage of true species tree topologies within the 95% credible set of topologies. (B) The average rooted Robinson-Foulds (RF) distance between the maximum clade credibility (MCC) species tree topology and the simulated true topology. Error bars are 95% confidence intervals calculated by bootstrapping. N = 30.

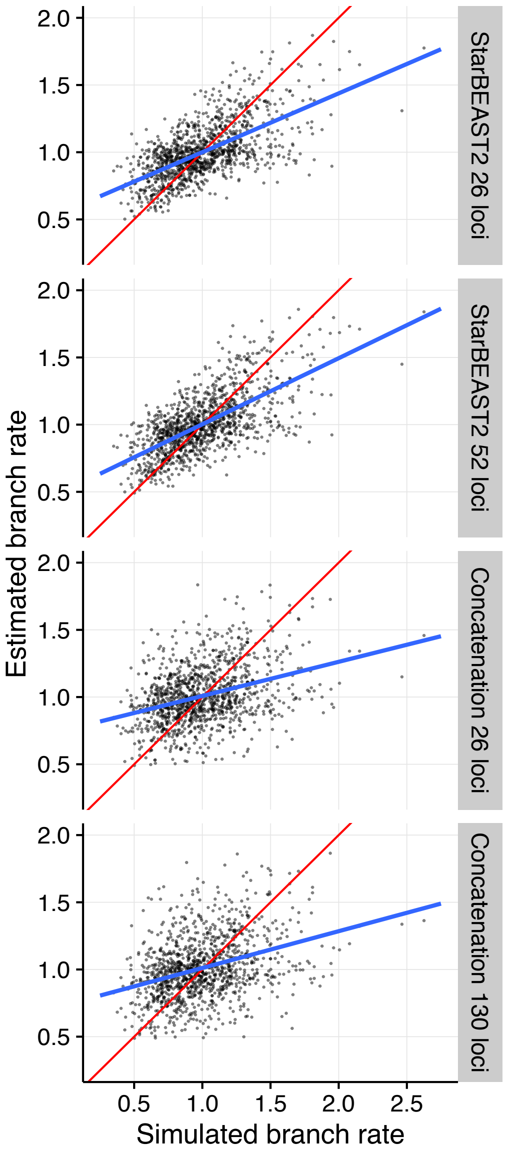

Figure S12: Estimates of species tree branch rates using unphased BEAST concatenation versus StarBEAST2. Estimated rates are the posterior expectations of each branch rate from each replicate. Root branch rates, which were fixed at 1, were excluded. In blue are simple linear regression lines of best fit, and in red are the $y = x$ lines showing a perfect relationship between estimates and truth. N = 30.

2 Supplementary tables

Table S1: Key for tested configurations.

Key	Method	Clock model	Co-ops	Popsizes	Loci	DNA
beast-1x-00	Concatenation	Strict	NA	NA	26	Phased
beast-1x-01	Concatenation	Strict	NA	NA	26	Unphased
beast-1x-20	Concatenation	ST-UCLN	NA	NA	26	Phased
beast-1x-21	Concatenation	ST-UCLN	NA	NA	26	Unphased
beast-5x-00	Concatenation	Strict	NA	NA	130	Phased
beast-5x-01	Concatenation	Strict	NA	NA	130	Unphased
beast-5x-20	Concatenation	ST-UCLN	NA	NA	130	Phased
beast-5x-21	Concatenation	ST-UCLN	NA	NA	130	Unphased
starbeast1-1x-00	*BEAST	Strict	Neither	MCMC	26 (50)	Phased
starbeast1-1x-10	*BEAST	GT-UCLN	Neither	MCMC	26 (50)	Phased
starbeast2-1x-00	StarBEAST2	Strict	Neither	MCMC	26	Phased
starbeast2-1x-01	StarBEAST2	Strict	Neither	Analytical	26	Phased
starbeast2-1x-02	StarBEAST2	Strict	Heights	MCMC	26	Phased
starbeast2-1x-03	StarBEAST2	Strict	Heights	Analytical	26 (50)	Phased
starbeast2-1x-04	StarBEAST2	Strict	Topology	MCMC	26	Phased
starbeast2-1x-05	StarBEAST2	Strict	Topology	Analytical	26	Phased
starbeast2-1x-06	StarBEAST2	Strict	Both	MCMC	26	Phased
starbeast2-1x-07	StarBEAST2	Strict	Both	Analytical	26	Phased
starbeast2-1x-10	StarBEAST2	GT-UCLN	Neither	MCMC	26	Phased
starbeast2-1x-11	StarBEAST2	GT-UCLN	Neither	Analytical	26	Phased
starbeast2-1x-12	StarBEAST2	GT-UCLN	Heights	MCMC	26	Phased
starbeast2-1x-13	StarBEAST2	GT-UCLN	Heights	Analytical	26 (50)	Phased
starbeast2-1x-14	StarBEAST2	GT-UCLN	Topology	MCMC	26	Phased
starbeast2-1x-15	StarBEAST2	GT-UCLN	Topology	Analytical	26	Phased
starbeast2-1x-16	StarBEAST2	GT-UCLN	Both	MCMC	26	Phased
starbeast2-1x-17	StarBEAST2	GT-UCLN	Both	Analytical	26	Phased
starbeast2-1x-20	StarBEAST2	ST-UCLN	Neither	MCMC	26	Phased
starbeast2-1x-21	StarBEAST2	ST-UCLN	Neither	Analytical	26	Phased
starbeast2-1x-22	StarBEAST2	ST-UCLN	Heights	MCMC	26	Phased
starbeast2-1x-23	StarBEAST2	ST-UCLN	Heights	Analytical	26 (50)	Phased
starbeast2-1x-24	StarBEAST2	ST-UCLN	Topology	MCMC	26	Phased
starbeast2-1x-25	StarBEAST2	ST-UCLN	Topology	Analytical	26	Phased
starbeast2-1x-26	StarBEAST2	ST-UCLN	Both	MCMC	26	Phased
starbeast2-1x-27	StarBEAST2	ST-UCLN	Both	Analytical	26	Phased
starbeast2-2x-03	StarBEAST2	Strict	Heights	Analytical	52 (100)	Phased
starbeast2-2x-13	StarBEAST2	GT-UCLN	Heights	Analytical	52 (100)	Phased
starbeast2-2x-23	StarBEAST2	ST-UCLN	Heights	Analytical	52 (100)	Phased

Loci numbers in brackets are for *Crocidura* reanalyses

Table S2: Ln(ESS per hour) convergence for simulated data.

key	extinctionfraction	likelihood	minimum	netdiversificationrate	popmean	posterior	prior	speciescoalescent	speciestree	treeheight
beast-1x-00	7.598 ± 0.135	6.129 ± 0.184	5.865 ± 0.197	7.904 ± 0.125	*NA*	5.967 ± 0.182	6.085 ± 0.390	*NA*	8.183 ± 0.137	8.214 ± 0.128
beast-1x-01	7.200 ± 0.369	5.736 ± 0.242	5.313 ± 0.288	7.486 ± 0.441	*NA*	5.482 ± 0.259	5.424 ± 0.381	*NA*	8.349 ± 0.339	8.347 ± 0.364
beast-1x-20	7.271 ± 0.260	5.969 ± 0.286	5.109 ± 0.374	7.252 ± 0.343	*NA*	5.745 ± 0.247	5.391 ± 0.310	*NA*	6.407 ± 0.403	5.335 ± 0.550
beast-1x-21	6.689 ± 0.325	5.717 ± 0.171	4.890 ± 0.358	6.691 ± 0.368	*NA*	5.457 ± 0.186	5.044 ± 0.268	*NA*	6.181 ± 0.417	5.116 ± 0.491
beast-5x-00	5.437 ± 0.145	3.679 ± 0.231	3.585 ± 0.248	5.865 ± 0.195	*NA*	3.618 ± 0.256	4.372 ± 0.478	*NA*	6.373 ± 0.222	6.754 ± 0.326
beast-5x-01	4.878 ± 0.289	3.305 ± 0.232	3.196 ± 0.256	5.163 ± 0.383	*NA*	3.204 ± 0.262	3.671 ± 0.398	*NA*	6.090 ± 0.367	6.302 ± 0.413
beast-5x-20	3.503 ± 0.782	2.976 ± 0.521	0.333 ± 0.868	3.051 ± 0.644	*NA*	2.894 ± 0.475	2.981 ± 0.517	*NA*	1.216 ± 0.549	0.333 ± 0.868
beast-5x-21	3.300 ± 0.632	2.743 ± 0.313	0.218 ± 0.617	2.929 ± 0.592	*NA*	2.602 ± 0.273	2.659 ± 0.347	*NA*	0.930 ± 0.551	0.220 ± 0.619
starbeast1-1x-00	6.123 ± 0.370	4.480 ± 0.341	3.716 ± 0.589	6.240 ± 0.498	5.224 ± 0.380	4.662 ± 0.808	4.179 ± 0.164	4.563 ± 0.779	6.533 ± 0.564	4.139 ± 0.956
starbeast1-1x-10	5.063 ± 0.532	4.339 ± 0.319	2.043 ± 0.447	4.135 ± 0.392	3.040 ± 0.275	2.368 ± 0.290	3.542 ± 0.312	2.345 ± 0.287	2.526 ± 0.300	2.059 ± 0.451
starbeast2-1x-00	6.624 ± 0.385	5.376 ± 0.405	4.828 ± 0.597	6.740 ± 0.433	5.703 ± 0.407	5.511 ± 0.917	5.223 ± 0.348	5.328 ± 0.906	7.487 ± 0.660	5.432 ± 1.023
starbeast2-1x-01	6.624 ± 0.378	5.494 ± 0.380	5.252 ± 0.545	6.702 ± 0.471	6.326 ± 0.509	6.672 ± 0.459	6.551 ± 0.149	6.589 ± 0.495	7.922 ± 0.511	5.695 ± 0.952
starbeast2-1x-02	6.421 ± 0.394	5.375 ± 0.351	4.845 ± 0.571	6.510 ± 0.450	5.613 ± 0.465	5.404 ± 0.919	5.090 ± 0.391	5.239 ± 0.944	7.546 ± 0.653	6.575 ± 0.762
starbeast2-1x-03	6.495 ± 0.360	5.762 ± 0.246	5.740 ± 0.238	6.663 ± 0.401	6.683 ± 0.311	6.955 ± 0.413	6.546 ± 0.190	6.882 ± 0.442	8.190 ± 0.419	7.670 ± 0.554
starbeast2-1x-04	6.198 ± 0.371	4.817 ± 0.518	4.270 ± 0.656	6.277 ± 0.446	5.198 ± 0.410	4.993 ± 0.988	4.663 ± 0.422	4.759 ± 0.963	6.992 ± 0.675	4.825 ± 0.895
starbeast2-1x-05	6.295 ± 0.362	5.264 ± 0.321	4.959 ± 0.591	6.409 ± 0.420	5.887 ± 0.527	6.262 ± 0.515	6.235 ± 0.177	6.107 ± 0.445	7.519 ± 0.601	5.486 ± 1.083
starbeast2-1x-06	6.099 ± 0.349	5.181 ± 0.219	4.532 ± 0.513	6.217 ± 0.411	5.267 ± 0.383	5.245 ± 0.895	4.763 ± 0.360	5.049 ± 0.863	7.313 ± 0.578	6.204 ± 0.759
starbeast2-1x-07	6.094 ± 0.401	5.525 ± 0.275	5.449 ± 0.326	6.192 ± 0.449	6.233 ± 0.420	6.674 ± 0.529	6.207 ± 0.212	6.560 ± 0.553	7.882 ± 0.415	7.319 ± 0.561
starbeast2-1x-10	6.286 ± 0.504	5.370 ± 0.307	3.930 ± 0.782	6.348 ± 0.531	5.275 ± 0.371	4.874 ± 0.840	4.807 ± 0.272	4.681 ± 0.820	6.655 ± 0.741	4.172 ± 1.166
starbeast2-1x-11	6.455 ± 0.487	5.474 ± 0.308	4.682 ± 0.864	6.503 ± 0.567	5.935 ± 0.580	6.185 ± 0.526	6.275 ± 0.268	6.143 ± 0.524	7.259 ± 0.856	4.994 ± 1.323
starbeast2-1x-12	6.285 ± 0.453	5.397 ± 0.246	4.614 ± 0.500	6.384 ± 0.495	5.390 ± 0.425	5.090 ± 0.817	4.866 ± 0.401	4.910 ± 0.810	7.282 ± 0.581	6.048 ± 0.745
starbeast2-1x-13	6.407 ± 0.390	5.598 ± 0.191	5.542 ± 0.243	6.514 ± 0.477	6.298 ± 0.509	6.490 ± 0.521	6.312 ± 0.199	6.407 ± 0.580	7.832 ± 0.445	6.987 ± 0.682
starbeast2-1x-14	5.862 ± 0.409	4.894 ± 0.368	3.566 ± 0.778	5.894 ± 0.451	4.830 ± 0.463	4.263 ± 0.923	4.417 ± 0.426	4.111 ± 0.919	6.250 ± 0.749	3.764 ± 1.140
starbeast2-1x-15	6.020 ± 0.489	5.001 ± 0.283	4.141 ± 0.799	6.136 ± 0.540	5.468 ± 0.515	5.669 ± 0.617	5.824 ± 0.227	5.547 ± 0.611	6.785 ± 0.710	4.370 ± 1.176
starbeast2-1x-16	6.070 ± 0.317	5.078 ± 0.253	4.238 ± 0.553	6.165 ± 0.399	5.058 ± 0.290	4.678 ± 0.826	4.608 ± 0.301	4.531 ± 0.829	6.961 ± 0.552	5.545 ± 0.802
starbeast2-1x-17	6.053 ± 0.310	5.308 ± 0.215	5.274 ± 0.238	6.164 ± 0.435	5.880 ± 0.325	6.195 ± 0.355	6.010 ± 0.229	6.109 ± 0.335	7.596 ± 0.375	6.759 ± 0.554
starbeast2-1x-20	3.968 ± 1.200	3.685 ± 0.706	1.176 ± 1.161	3.871 ± 1.199	3.560 ± 0.633	2.858 ± 0.774	3.018 ± 0.665	2.747 ± 0.754	3.061 ± 1.068	1.193 ± 1.169
starbeast2-1x-21	4.111 ± 1.292	3.845 ± 0.868	1.450 ± 1.442	3.876 ± 1.307	3.732 ± 0.918	3.294 ± 0.982	3.823 ± 1.069	3.304 ± 0.953	3.189 ± 1.207	1.463 ± 1.473
starbeast2-1x-22	5.318 ± 0.431	4.670 ± 0.427	3.223 ± 0.548	5.124 ± 0.492	4.420 ± 0.287	3.881 ± 0.600	3.809 ± 0.308	3.772 ± 0.615	4.366 ± 0.460	3.343 ± 0.580
starbeast2-1x-23	5.448 ± 0.329	4.968 ± 0.194	3.712 ± 0.666	5.339 ± 0.398	4.897 ± 0.328	4.442 ± 0.412	4.983 ± 0.469	4.449 ± 0.402	4.566 ± 0.527	3.725 ± 0.681
starbeast2-1x-24	3.629 ± 1.388	3.412 ± 0.839	0.912 ± 1.398	3.473 ± 1.366	3.356 ± 0.799	2.668 ± 0.831	2.823 ± 0.718	2.582 ± 0.822	2.771 ± 1.173	0.921 ± 1.417
starbeast2-1x-25	3.653 ± 1.292	3.567 ± 0.853	0.899 ± 1.415	3.466 ± 1.320	3.512 ± 0.913	3.157 ± 0.936	3.432 ± 1.115	3.130 ± 0.936	2.800 ± 1.242	0.925 ± 1.477
starbeast2-1x-26	5.075 ± 0.514	4.435 ± 0.378	2.981 ± 0.726	4.894 ± 0.580	4.194 ± 0.299	3.687 ± 0.565	3.576 ± 0.349	3.592 ± 0.614	4.169 ± 0.564	3.106 ± 0.754
starbeast2-1x-27	5.273 ± 0.455	4.785 ± 0.267	3.429 ± 0.649	5.100 ± 0.526	4.815 ± 0.393	4.316 ± 0.350	4.880 ± 0.466	4.301 ± 0.351	4.452 ± 0.575	3.440 ± 0.666
starbeast2-2x-03	5.732 ± 0.280	4.266 ± 0.282	4.226 ± 0.256	5.816 ± 0.366	4.969 ± 0.454	5.245 ± 0.546	5.239 ± 0.237	5.164 ± 0.507	6.828 ± 0.442	5.954 ± 0.518
starbeast2-2x-13	5.640 ± 0.424	4.221 ± 0.206	4.178 ± 0.197	5.729 ± 0.498	4.788 ± 0.492	5.016 ± 0.418	5.111 ± 0.216	4.958 ± 0.453	6.629 ± 0.359	5.386 ± 0.573
starbeast2-2x-23	3.932 ± 0.643	3.263 ± 0.311	1.169 ± 0.737	3.579 ± 0.687	2.869 ± 0.416	2.079 ± 0.439	3.618 ± 0.544	2.077 ± 0.414	2.161 ± 0.614	1.173 ± 0.742

All values are mean(Ln(ESS rate)) ± sd(Ln(ESS rate))
The configuration for each key is described by Table S1

Table S3: Ln(ESS per million states) convergence for simulated data.

key	extinctionfraction	likelihood	minimum	netdiversificationrate	popmean	posterior	prior	speciescoalescent	speciestree	treeheight
beast-1x-00	6.223 ± 0.103	4.754 ± 0.174	4.490 ± 0.244	6.528 ± 0.131	*NA*	4.591 ± 0.195	4.710 ± 0.430	*NA*	6.808 ± 0.061	6.839 ± 0.054
beast-1x-01	6.180 ± 0.346	4.716 ± 0.228	4.294 ± 0.294	6.467 ± 0.420	*NA*	4.462 ± 0.280	4.404 ± 0.390	*NA*	7.329 ± 0.293	7.328 ± 0.355
beast-1x-20	5.886 ± 0.249	4.584 ± 0.286	3.724 ± 0.334	5.867 ± 0.299	*NA*	4.360 ± 0.261	4.006 ± 0.271	*NA*	5.022 ± 0.312	3.950 ± 0.497
beast-1x-21	5.626 ± 0.323	4.653 ± 0.186	3.827 ± 0.340	5.628 ± 0.355	*NA*	4.394 ± 0.214	3.981 ± 0.255	*NA*	5.118 ± 0.341	4.053 ± 0.461
beast-5x-00	5.623 ± 0.134	3.865 ± 0.218	3.772 ± 0.244	6.051 ± 0.190	*NA*	3.805 ± 0.258	4.558 ± 0.533	*NA*	6.560 ± 0.196	6.940 ± 0.305
beast-5x-01	5.438 ± 0.302	3.865 ± 0.265	3.756 ± 0.294	5.723 ± 0.407	*NA*	3.765 ± 0.299	4.232 ± 0.452	*NA*	6.650 ± 0.395	6.862 ± 0.447
beast-5x-20	3.730 ± 0.767	3.204 ± 0.540	0.560 ± 0.851	3.279 ± 0.624	*NA*	3.121 ± 0.497	3.208 ± 0.505	*NA*	1.443 ± 0.506	0.560 ± 0.851
beast-5x-21	3.890 ± 0.610	3.333 ± 0.308	0.808 ± 0.582	3.519 ± 0.572	*NA*	3.192 ± 0.282	3.249 ± 0.343	*NA*	1.520 ± 0.513	0.810 ± 0.584
starbeast1-1x-00	3.654 ± 0.401	2.010 ± 0.352	1.247 ± 0.601	3.770 ± 0.531	2.755 ± 0.394	2.193 ± 0.822	1.710 ± 0.141	2.094 ± 0.791	4.064 ± 0.595	1.670 ± 0.968
starbeast1-1x-10	2.575 ± 0.522	1.851 ± 0.323	−0.446 ± 0.425	1.646 ± 0.369	0.551 ± 0.227	−0.121 ± 0.245	1.053 ± 0.282	−0.144 ± 0.241	0.037 ± 0.250	−0.429 ± 0.430
starbeast2-1x-00	3.627 ± 0.396	2.379 ± 0.390	1.831 ± 0.589	3.744 ± 0.440	2.707 ± 0.399	2.514 ± 0.913	2.227 ± 0.336	2.331 ± 0.900	4.490 ± 0.661	2.435 ± 1.026
starbeast2-1x-01	3.737 ± 0.378	2.607 ± 0.378	2.365 ± 0.559	3.815 ± 0.471	3.439 ± 0.512	3.785 ± 0.467	3.664 ± 0.179	3.702 ± 0.507	5.035 ± 0.526	2.808 ± 0.981
starbeast2-1x-02	3.558 ± 0.401	2.512 ± 0.361	1.981 ± 0.586	3.647 ± 0.461	2.750 ± 0.476	2.540 ± 0.930	2.226 ± 0.392	2.376 ± 0.951	4.683 ± 0.665	3.712 ± 0.782
starbeast2-1x-03	3.703 ± 0.350	2.970 ± 0.219	2.948 ± 0.207	3.872 ± 0.396	3.891 ± 0.328	4.163 ± 0.424	3.754 ± 0.192	4.091 ± 0.458	5.398 ± 0.419	4.878 ± 0.549
starbeast2-1x-04	3.628 ± 0.370	2.247 ± 0.533	1.701 ± 0.656	3.708 ± 0.449	2.628 ± 0.385	2.423 ± 0.984	2.094 ± 0.397	2.190 ± 0.961	4.423 ± 0.678	2.255 ± 0.910
starbeast2-1x-05	3.797 ± 0.363	2.766 ± 0.301	2.461 ± 0.585	3.911 ± 0.428	3.389 ± 0.521	3.764 ± 0.520	3.737 ± 0.185	3.609 ± 0.438	5.020 ± 0.598	2.988 ± 1.065
starbeast2-1x-06	3.565 ± 0.357	2.648 ± 0.223	1.998 ± 0.529	3.683 ± 0.421	2.733 ± 0.382	2.711 ± 0.904	2.230 ± 0.352	2.515 ± 0.879	4.779 ± 0.593	3.670 ± 0.798
starbeast2-1x-07	3.631 ± 0.401	3.061 ± 0.249	2.985 ± 0.300	3.728 ± 0.454	3.770 ± 0.412	4.211 ± 0.517	3.743 ± 0.235	4.096 ± 0.541	5.419 ± 0.391	4.855 ± 0.560
starbeast2-1x-10	3.322 ± 0.499	2.406 ± 0.308	0.966 ± 0.777	3.384 ± 0.526	2.311 ± 0.377	1.910 ± 0.834	1.843 ± 0.273	1.718 ± 0.815	3.691 ± 0.738	1.208 ± 1.166
starbeast2-1x-11	3.588 ± 0.506	2.606 ± 0.321	1.815 ± 0.888	3.635 ± 0.581	3.068 ± 0.602	3.318 ± 0.543	3.408 ± 0.293	3.276 ± 0.542	4.391 ± 0.880	2.127 ± 1.340
starbeast2-1x-12	3.444 ± 0.447	2.555 ± 0.250	1.773 ± 0.487	3.543 ± 0.491	2.548 ± 0.416	2.248 ± 0.804	2.024 ± 0.360	2.069 ± 0.797	4.441 ± 0.580	3.207 ± 0.751
starbeast2-1x-13	3.641 ± 0.402	2.832 ± 0.190	2.776 ± 0.236	3.747 ± 0.491	3.532 ± 0.489	3.724 ± 0.498	3.546 ± 0.224	3.641 ± 0.551	5.066 ± 0.441	4.221 ± 0.670
starbeast2-1x-14	3.312 ± 0.419	2.344 ± 0.379	1.016 ± 0.780	3.344 ± 0.468	2.279 ± 0.466	1.713 ± 0.921	1.867 ± 0.417	1.561 ± 0.913	3.700 ± 0.755	1.213 ± 1.139
starbeast2-1x-15	3.562 ± 0.489	2.543 ± 0.289	1.683 ± 0.814	3.678 ± 0.540	3.010 ± 0.534	3.211 ± 0.631	3.366 ± 0.246	3.089 ± 0.625	4.327 ± 0.725	1.912 ± 1.183
starbeast2-1x-16	3.555 ± 0.323	2.564 ± 0.251	1.723 ± 0.574	3.651 ± 0.407	2.543 ± 0.289	2.163 ± 0.839	2.093 ± 0.305	2.017 ± 0.844	4.447 ± 0.570	3.031 ± 0.817
starbeast2-1x-17	3.621 ± 0.314	2.877 ± 0.201	2.843 ± 0.236	3.732 ± 0.447	3.449 ± 0.327	3.763 ± 0.352	3.579 ± 0.217	3.677 ± 0.346	5.165 ± 0.393	4.327 ± 0.568
starbeast2-1x-20	1.814 ± 1.221	1.531 ± 0.715	−0.978 ± 1.179	1.717 ± 1.217	1.406 ± 0.647	0.705 ± 0.797	0.864 ± 0.655	0.593 ± 0.775	0.908 ± 1.064	−0.961 ± 1.188
starbeast2-1x-21	2.016 ± 1.272	1.750 ± 0.844	−0.646 ± 1.418	1.781 ± 1.290	1.637 ± 0.890	1.199 ± 0.966	1.728 ± 1.034	1.208 ± 0.934	1.094 ± 1.185	−0.633 ± 1.448
starbeast2-1x-22	3.156 ± 0.428	2.508 ± 0.428	1.061 ± 0.544	2.962 ± 0.482	2.258 ± 0.279	1.718 ± 0.607	1.647 ± 0.281	1.610 ± 0.621	2.204 ± 0.438	1.181 ± 0.569
starbeast2-1x-23	3.368 ± 0.317	2.889 ± 0.209	1.633 ± 0.666	3.259 ± 0.386	2.818 ± 0.300	2.362 ± 0.413	2.903 ± 0.446	2.370 ± 0.403	2.487 ± 0.524	1.646 ± 0.681
starbeast2-1x-24	1.682 ± 1.382	1.464 ± 0.836	−1.035 ± 1.382	1.525 ± 1.353	1.409 ± 0.785	0.720 ± 0.820	0.875 ± 0.694	0.634 ± 0.812	0.823 ± 1.153	−1.027 ± 1.401
starbeast2-1x-25	1.766 ± 1.285	1.679 ± 0.838	−0.989 ± 1.400	1.578 ± 1.312	1.624 ± 0.899	1.269 ± 0.928	1.544 ± 1.090	1.243 ± 0.927	0.912 ± 1.225	−0.963 ± 1.463
starbeast2-1x-26	3.081 ± 0.495	2.441 ± 0.356	0.987 ± 0.694	2.900 ± 0.558	2.199 ± 0.261	1.693 ± 0.567	1.582 ± 0.304	1.598 ± 0.615	2.175 ± 0.524	1.111 ± 0.717
starbeast2-1x-27	3.337 ± 0.435	2.850 ± 0.238	1.494 ± 0.612	3.164 ± 0.500	2.879 ± 0.347	2.381 ± 0.293	2.945 ± 0.418	2.365 ± 0.300	2.516 ± 0.530	1.505 ± 0.627
starbeast2-2x-03	3.649 ± 0.283	2.182 ± 0.299	2.143 ± 0.274	3.733 ± 0.379	2.885 ± 0.460	3.162 ± 0.565	3.156 ± 0.257	3.081 ± 0.530	4.744 ± 0.460	3.871 ± 0.522
starbeast2-2x-13	3.563 ± 0.433	2.143 ± 0.231	2.100 ± 0.217	3.652 ± 0.505	2.711 ± 0.491	2.939 ± 0.423	3.033 ± 0.247	2.881 ± 0.451	4.552 ± 0.365	3.309 ± 0.568
starbeast2-2x-23	2.536 ± 0.625	1.868 ± 0.296	−0.226 ± 0.719	2.183 ± 0.667	1.473 ± 0.396	0.683 ± 0.425	2.222 ± 0.515	0.681 ± 0.402	0.766 ± 0.580	−0.223 ± 0.723

All values are mean(Ln(ESS rate)) ± sd(Ln(ESS rate))
The configuration for each key is described by Table S1

Table S4: Ln(ESS per hour) convergence for *Pseudacris* chorus frogs.

key	extinctionfraction	likelihood	minimum	netdiversificationrate	popmean	posterior	prior	speciescoalescent	speciestree	treeheight
starbeast1-1x-00	5.598 ± 0.637	2.862 ± 0.411	2.814 ± 0.451	5.689 ± 0.669	4.664 ± 0.462	4.185 ± 0.495	3.415 ± 0.397	3.906 ± 0.441	5.647 ± 0.723	3.915 ± 0.481
starbeast1-1x-10	4.560 ± 0.903	2.409 ± 0.411	1.376 ± 1.614	3.927 ± 0.705	2.933 ± 0.394	2.190 ± 1.202	2.289 ± 1.843	2.315 ± 0.291	2.610 ± 0.301	1.756 ± 0.392
starbeast2-1x-00	6.343 ± 0.239	3.824 ± 0.502	3.804 ± 0.486	6.409 ± 0.268	5.122 ± 0.282	4.673 ± 0.360	4.914 ± 0.240	4.381 ± 0.320	6.629 ± 0.514	4.883 ± 0.371
starbeast2-1x-01	6.298 ± 0.245	3.928 ± 0.376	3.925 ± 0.376	6.369 ± 0.269	5.092 ± 0.435	5.552 ± 0.488	5.887 ± 0.172	5.191 ± 0.546	6.732 ± 0.442	4.753 ± 0.432
starbeast2-1x-02	6.165 ± 0.200	3.882 ± 0.409	3.878 ± 0.412	6.260 ± 0.209	5.230 ± 0.289	4.949 ± 0.489	4.842 ± 0.183	4.561 ± 0.485	6.791 ± 0.463	6.270 ± 0.274
starbeast2-1x-03	6.232 ± 0.208	4.159 ± 0.384	4.159 ± 0.384	6.337 ± 0.235	5.691 ± 0.274	6.044 ± 0.297	5.832 ± 0.195	5.622 ± 0.365	6.971 ± 0.458	6.462 ± 0.318
starbeast2-1x-04	5.917 ± 0.243	3.309 ± 0.326	3.297 ± 0.339	5.999 ± 0.236	4.710 ± 0.320	4.453 ± 0.548	4.500 ± 0.250	4.080 ± 0.552	6.175 ± 0.550	4.479 ± 0.444
starbeast2-1x-05	5.926 ± 0.407	3.749 ± 0.475	3.739 ± 0.482	6.011 ± 0.409	5.079 ± 0.380	5.415 ± 0.442	5.579 ± 0.174	5.125 ± 0.432	6.557 ± 0.559	4.431 ± 0.521
starbeast2-1x-06	5.878 ± 0.261	3.631 ± 0.379	3.622 ± 0.352	6.002 ± 0.282	4.878 ± 0.256	4.688 ± 0.422	4.508 ± 0.172	4.315 ± 0.413	6.534 ± 0.463	5.973 ± 0.277
starbeast2-1x-07	5.856 ± 0.266	3.827 ± 0.415	3.827 ± 0.415	5.996 ± 0.241	5.406 ± 0.292	5.796 ± 0.270	5.598 ± 0.121	5.404 ± 0.262	6.704 ± 0.377	6.166 ± 0.227
starbeast2-1x-10	5.625 ± 0.446	3.414 ± 0.281	2.843 ± 0.433	5.587 ± 0.472	4.475 ± 0.333	3.950 ± 0.371	4.318 ± 0.291	3.681 ± 0.346	5.247 ± 0.453	2.859 ± 0.453
starbeast2-1x-11	5.627 ± 0.457	3.494 ± 0.376	2.774 ± 0.568	5.610 ± 0.516	4.606 ± 0.426	4.693 ± 0.437	5.117 ± 0.211	4.451 ± 0.418	5.268 ± 0.581	2.788 ± 0.575
starbeast2-1x-12	6.113 ± 0.221	3.912 ± 0.431	3.842 ± 0.398	6.153 ± 0.214	5.016 ± 0.345	4.560 ± 0.591	4.670 ± 0.223	4.275 ± 0.601	6.305 ± 0.483	4.542 ± 0.501
starbeast2-1x-13	6.115 ± 0.224	3.981 ± 0.359	3.981 ± 0.359	6.198 ± 0.237	5.464 ± 0.307	5.596 ± 0.286	5.664 ± 0.141	5.209 ± 0.331	6.457 ± 0.295	4.787 ± 0.273
starbeast2-1x-14	5.437 ± 0.374	3.190 ± 0.366	2.675 ± 0.500	5.435 ± 0.425	4.219 ± 0.447	3.701 ± 0.534	3.903 ± 0.379	3.418 ± 0.538	5.159 ± 0.482	2.715 ± 0.549
starbeast2-1x-15	5.216 ± 0.420	3.106 ± 0.249	2.360 ± 0.408	5.172 ± 0.404	4.180 ± 0.249	4.373 ± 0.409	4.767 ± 0.216	3.974 ± 0.313	4.860 ± 0.489	2.368 ± 0.423
starbeast2-1x-16	5.766 ± 0.291	3.446 ± 0.389	3.411 ± 0.388	5.786 ± 0.315	4.584 ± 0.284	4.135 ± 0.447	4.287 ± 0.238	3.839 ± 0.478	5.811 ± 0.446	4.194 ± 0.337
starbeast2-1x-17	5.809 ± 0.249	3.684 ± 0.437	3.661 ± 0.388	5.866 ± 0.216	5.136 ± 0.315	5.218 ± 0.277	5.380 ± 0.185	4.902 ± 0.365	6.110 ± 0.359	4.427 ± 0.213
starbeast2-1x-20	3.974 ± 0.944	2.712 ± 0.378	1.340 ± 0.906	3.806 ± 0.956	3.575 ± 0.524	2.919 ± 0.547	3.441 ± 0.434	2.714 ± 0.537	3.153 ± 0.958	1.340 ± 0.906
starbeast2-1x-21	4.646 ± 0.736	3.086 ± 0.325	2.054 ± 0.956	4.586 ± 0.734	4.076 ± 0.489	3.639 ± 0.597	4.377 ± 0.574	3.543 ± 0.590	3.849 ± 0.752	2.109 ± 1.040
starbeast2-1x-22	5.183 ± 0.237	3.088 ± 0.346	3.046 ± 0.315	5.072 ± 0.262	4.300 ± 0.161	3.768 ± 0.285	3.891 ± 0.214	3.528 ± 0.292	4.571 ± 0.216	3.503 ± 0.306
starbeast2-1x-23	5.324 ± 0.275	3.446 ± 0.305	3.444 ± 0.300	5.264 ± 0.249	4.687 ± 0.262	4.419 ± 0.259	4.894 ± 0.196	4.268 ± 0.284	4.793 ± 0.214	3.976 ± 0.245
starbeast2-1x-24	4.047 ± 0.704	2.597 ± 0.260	1.264 ± 0.767	3.840 ± 0.769	3.526 ± 0.368	2.764 ± 0.376	3.374 ± 0.273	2.573 ± 0.362	3.129 ± 0.844	1.281 ± 0.801
starbeast2-1x-25	4.096 ± 0.870	2.771 ± 0.331	1.525 ± 0.932	3.950 ± 0.907	3.669 ± 0.616	3.124 ± 0.829	3.988 ± 0.604	2.990 ± 0.750	3.364 ± 1.035	1.568 ± 1.020
starbeast2-1x-26	5.031 ± 0.232	2.894 ± 0.345	2.878 ± 0.332	4.939 ± 0.277	4.103 ± 0.272	3.619 ± 0.360	3.698 ± 0.200	3.357 ± 0.388	4.436 ± 0.283	3.455 ± 0.290
starbeast2-1x-27	5.169 ± 0.219	3.250 ± 0.278	3.243 ± 0.263	5.111 ± 0.227	4.507 ± 0.241	4.223 ± 0.245	4.721 ± 0.136	4.098 ± 0.257	4.617 ± 0.216	3.786 ± 0.220

All values are mean(Ln(ESS rate)) ± sd(Ln(ESS rate))
The configuration for each key is described by Table S1

Table S5: Ln(ESS per million states) convergence for *Pseudacris* chorus frogs.

key	extinctionfraction	likelihood	minimum	netdiversificationrate	popmean	posterior	prior	speciescoalescent	speciestree	treeheight
starbeast1-1x-00	3.216 ± 0.635	0.480 ± 0.409	0.432 ± 0.450	3.307 ± 0.666	2.282 ± 0.455	1.803 ± 0.496	1.033 ± 0.401	1.524 ± 0.439	3.265 ± 0.722	1.533 ± 0.476
starbeast1-1x-10	2.153 ± 0.898	0.001 ± 0.408	-1.032 ± 1.608	1.519 ± 0.700	0.525 ± 0.389	-0.217 ± 1.198	-0.118 ± 1.837	-0.092 ± 0.287	0.202 ± 0.295	-0.652 ± 0.390
starbeast2-1x-00	3.446 ± 0.235	0.926 ± 0.504	0.906 ± 0.488	3.511 ± 0.264	2.225 ± 0.281	1.775 ± 0.364	2.017 ± 0.239	1.483 ± 0.325	3.732 ± 0.515	1.985 ± 0.371
starbeast2-1x-01	3.514 ± 0.242	1.145 ± 0.370	1.141 ± 0.370	3.585 ± 0.263	2.308 ± 0.438	2.768 ± 0.491	3.104 ± 0.157	2.407 ± 0.548	3.949 ± 0.440	1.970 ± 0.428
starbeast2-1x-02	3.395 ± 0.191	1.112 ± 0.404	1.108 ± 0.406	3.490 ± 0.202	2.460 ± 0.282	2.179 ± 0.484	2.072 ± 0.176	1.791 ± 0.479	4.021 ± 0.458	3.500 ± 0.270
starbeast2-1x-03	3.566 ± 0.187	1.493 ± 0.362	1.493 ± 0.362	3.671 ± 0.211	3.025 ± 0.259	3.378 ± 0.274	3.166 ± 0.176	2.956 ± 0.341	4.305 ± 0.438	3.795 ± 0.300
starbeast2-1x-04	3.418 ± 0.252	0.810 ± 0.322	0.798 ± 0.336	3.500 ± 0.243	2.210 ± 0.316	1.954 ± 0.549	2.001 ± 0.243	1.581 ± 0.553	3.676 ± 0.550	1.980 ± 0.441
starbeast2-1x-05	3.506 ± 0.401	1.329 ± 0.475	1.319 ± 0.483	3.592 ± 0.405	2.659 ± 0.373	2.996 ± 0.445	3.160 ± 0.165	2.706 ± 0.438	4.138 ± 0.562	2.012 ± 0.530
starbeast2-1x-06	3.403 ± 0.253	1.155 ± 0.370	1.146 ± 0.343	3.526 ± 0.274	2.403 ± 0.262	2.212 ± 0.424	2.033 ± 0.168	1.839 ± 0.415	4.059 ± 0.453	3.497 ± 0.267
starbeast2-1x-07	3.465 ± 0.253	1.437 ± 0.406	1.437 ± 0.406	3.605 ± 0.229	3.016 ± 0.290	3.406 ± 0.274	3.208 ± 0.124	3.013 ± 0.265	4.314 ± 0.365	3.775 ± 0.217
starbeast2-1x-10	2.748 ± 0.443	0.537 ± 0.279	-0.034 ± 0.433	2.710 ± 0.469	1.599 ± 0.333	1.073 ± 0.378	1.441 ± 0.287	0.805 ± 0.353	2.371 ± 0.449	-0.018 ± 0.453
starbeast2-1x-11	2.848 ± 0.453	0.715 ± 0.373	-0.005 ± 0.564	2.831 ± 0.511	1.827 ± 0.428	1.914 ± 0.439	2.338 ± 0.207	1.671 ± 0.421	2.489 ± 0.579	0.009 ± 0.572
starbeast2-1x-12	3.357 ± 0.212	1.156 ± 0.422	1.087 ± 0.388	3.397 ± 0.207	2.260 ± 0.332	1.804 ± 0.580	1.914 ± 0.208	1.519 ± 0.594	3.549 ± 0.471	1.786 ± 0.493
starbeast2-1x-13	3.444 ± 0.215	1.310 ± 0.341	1.310 ± 0.341	3.528 ± 0.230	2.793 ± 0.300	2.925 ± 0.282	2.993 ± 0.137	2.538 ± 0.323	3.786 ± 0.284	2.116 ± 0.269
starbeast2-1x-14	2.951 ± 0.377	0.703 ± 0.358	0.189 ± 0.500	2.948 ± 0.427	1.733 ± 0.447	1.214 ± 0.539	1.416 ± 0.380	0.932 ± 0.543	2.673 ± 0.483	0.229 ± 0.549
starbeast2-1x-15	2.802 ± 0.418	0.692 ± 0.253	-0.054 ± 0.405	2.758 ± 0.401	1.766 ± 0.248	1.958 ± 0.409	2.353 ± 0.216	1.560 ± 0.312	2.446 ± 0.485	-0.046 ± 0.420
starbeast2-1x-16	3.311 ± 0.282	0.990 ± 0.383	0.955 ± 0.382	3.331 ± 0.311	2.128 ± 0.277	1.679 ± 0.443	1.832 ± 0.232	1.383 ± 0.472	3.356 ± 0.438	1.739 ± 0.333
starbeast2-1x-17	3.421 ± 0.248	1.296 ± 0.442	1.272 ± 0.392	3.477 ± 0.213	2.747 ± 0.315	2.829 ± 0.275	2.992 ± 0.181	2.514 ± 0.366	3.722 ± 0.360	2.038 ± 0.201
starbeast2-1x-20	1.926 ± 0.933	0.663 ± 0.367	-0.708 ± 0.895	1.758 ± 0.945	1.527 ± 0.513	0.870 ± 0.539	1.393 ± 0.424	0.666 ± 0.527	1.104 ± 0.947	-0.708 ± 0.895
starbeast2-1x-21	2.658 ± 0.731	1.098 ± 0.323	0.066 ± 0.955	2.598 ± 0.731	2.089 ± 0.485	1.651 ± 0.591	2.389 ± 0.568	1.556 ± 0.587	1.862 ± 0.749	0.122 ± 1.039
starbeast2-1x-22	3.119 ± 0.236	1.024 ± 0.348	0.982 ± 0.317	3.008 ± 0.265	2.236 ± 0.165	1.704 ± 0.277	1.827 ± 0.219	1.463 ± 0.287	2.507 ± 0.222	1.439 ± 0.300
starbeast2-1x-23	3.309 ± 0.273	1.431 ± 0.303	1.428 ± 0.297	3.248 ± 0.250	2.672 ± 0.255	2.403 ± 0.253	2.879 ± 0.190	2.252 ± 0.281	2.777 ± 0.202	1.960 ± 0.241
starbeast2-1x-24	2.200 ± 0.699	0.750 ± 0.262	-0.583 ± 0.761	1.993 ± 0.765	1.679 ± 0.365	0.917 ± 0.371	1.527 ± 0.268	0.726 ± 0.359	1.282 ± 0.839	-0.566 ± 0.794
starbeast2-1x-25	2.305 ± 0.854	0.980 ± 0.318	-0.267 ± 0.916	2.159 ± 0.891	1.878 ± 0.600	1.333 ± 0.815	2.197 ± 0.588	1.199 ± 0.736	1.573 ± 1.019	-0.223 ± 1.004
starbeast2-1x-26	3.147 ± 0.233	1.010 ± 0.344	0.994 ± 0.331	3.055 ± 0.272	2.219 ± 0.274	1.736 ± 0.360	1.814 ± 0.204	1.473 ± 0.390	2.552 ± 0.285	1.571 ± 0.294
starbeast2-1x-27	3.336 ± 0.205	1.417 ± 0.260	1.409 ± 0.245	3.278 ± 0.218	2.674 ± 0.231	2.390 ± 0.237	2.888 ± 0.131	2.265 ± 0.246	2.784 ± 0.199	1.953 ± 0.207

All values are mean(Ln(ESS rate)) ± sd(Ln(ESS rate))
The configuration for each key is described by Table S1

Table S6: Ln(ESS per hour) convergence for *Crocidura* shrews.

key	extinctionfraction	likelihood	minimum	netdiversificationrate	popmean	posterior	prior	speciescoalescent	speciestree	treeheight
starbeast1-1x-00	3.625 ± 0.592	2.438 ± 0.573	0.488 ± 0.548	3.616 ± 0.597	2.611 ± 0.410	0.735 ± 0.444	3.160 ± 0.320	0.759 ± 0.438	2.774 ± 0.557	0.845 ± 0.727
starbeast1-1x-10	3.327 ± 0.572	2.292 ± 0.456	0.165 ± 0.509	3.282 ± 0.569	2.320 ± 0.495	0.464 ± 0.429	2.736 ± 0.242	0.502 ± 0.414	2.589 ± 0.510	0.441 ± 0.698
starbeast2-1x-03	5.972 ± 0.511	4.025 ± 0.661	2.963 ± 0.458	5.954 ± 0.526	3.357 ± 0.382	2.993 ± 0.440	5.200 ± 0.166	2.986 ± 0.436	5.181 ± 0.409	4.545 ± 0.363
starbeast2-1x-13	5.863 ± 0.462	4.029 ± 0.478	2.793 ± 0.483	5.835 ± 0.469	3.250 ± 0.361	2.818 ± 0.475	4.730 ± 0.139	2.801 ± 0.481	5.021 ± 0.477	4.349 ± 0.419
starbeast2-1x-23	5.181 ± 0.445	3.215 ± 0.669	2.118 ± 0.509	4.946 ± 0.341	2.538 ± 0.532	2.201 ± 0.492	4.354 ± 0.187	2.183 ± 0.488	3.838 ± 0.403	2.811 ± 0.707
starbeast2-2x-03	3.993 ± 0.635	2.614 ± 0.455	0.832 ± 0.481	3.999 ± 0.626	1.522 ± 0.442	0.836 ± 0.482	3.564 ± 0.350	0.858 ± 0.477	3.642 ± 0.606	2.740 ± 0.321
starbeast2-2x-13	3.732 ± 0.724	2.342 ± 0.652	0.614 ± 0.536	3.710 ± 0.715	1.318 ± 0.446	0.622 ± 0.536	3.111 ± 0.316	0.638 ± 0.536	3.333 ± 0.655	2.418 ± 0.377
starbeast2-2x-23	2.707 ± 0.339	1.548 ± 0.425	0.166 ± 0.484	2.654 ± 0.311	0.736 ± 0.461	0.190 ± 0.492	2.710 ± 0.344	0.207 ± 0.485	2.077 ± 0.414	1.027 ± 0.650

All values are mean(Ln(ESS rate)) ± sd(Ln(ESS rate))
The configuration for each key is described by Table S1

Table S7: Ln(ESS per million states) convergence for *Crocidura* shrews.

key	extinctionfraction	likelihood	minimum	netdiversificationrate	popmean	posterior	prior	speciescoalescent	speciestree	treeheight
starbeast1-1x-00	0.775 ± 0.580	-0.413 ± 0.567	-2.363 ± 0.538	0.765 ± 0.585	-0.240 ± 0.400	-2.116 ± 0.433	0.309 ± 0.307	-2.092 ± 0.427	-0.077 ± 0.547	-2.006 ± 0.721
starbeast1-1x-10	0.487 ± 0.560	-0.548 ± 0.450	-2.676 ± 0.500	0.442 ± 0.557	-0.521 ± 0.487	-2.377 ± 0.418	-0.105 ± 0.235	-2.339 ± 0.403	-0.251 ± 0.500	-2.400 ± 0.693
starbeast2-1x-03	3.009 ± 0.514	1.063 ± 0.660	0.001 ± 0.460	2.992 ± 0.529	0.394 ± 0.383	0.031 ± 0.442	2.237 ± 0.170	0.024 ± 0.437	2.219 ± 0.410	1.583 ± 0.361
starbeast2-1x-13	2.906 ± 0.456	1.072 ± 0.479	-0.164 ± 0.477	2.878 ± 0.464	0.293 ± 0.354	-0.139 ± 0.470	1.773 ± 0.137	-0.156 ± 0.475	2.064 ± 0.472	1.392 ± 0.412
starbeast2-1x-23	3.071 ± 0.433	1.106 ± 0.666	0.008 ± 0.500	2.836 ± 0.328	0.429 ± 0.522	0.092 ± 0.482	2.244 ± 0.168	0.073 ± 0.478	1.729 ± 0.397	0.702 ± 0.697
starbeast2-2x-03	1.752 ± 0.619	0.373 ± 0.453	-1.409 ± 0.468	1.757 ± 0.610	-0.719 ± 0.432	-1.405 ± 0.469	1.322 ± 0.335	-1.383 ± 0.464	1.401 ± 0.593	0.499 ± 0.312
starbeast2-2x-13	1.505 ± 0.706	0.115 ± 0.644	-1.613 ± 0.520	1.483 ± 0.697	-0.909 ± 0.431	-1.605 ± 0.519	0.885 ± 0.300	-1.589 ± 0.519	1.106 ± 0.637	0.192 ± 0.363
starbeast2-2x-23	1.357 ± 0.337	0.198 ± 0.420	-1.184 ± 0.483	1.303 ± 0.309	-0.615 ± 0.456	-1.161 ± 0.491	1.360 ± 0.341	-1.144 ± 0.484	0.726 ± 0.410	-0.324 ± 0.648

All values are mean(Ln(ESS rate)) ± sd(Ln(ESS rate))
The configuration for each key is described by Table S1

Table S8: Initial chain lengths and sampling rates.

Methods	Initial chain length	Sampling rate (one per)
Concatenation 26 loci phased	$2^{23} = 8388608$	$2^{10} = 1024$
Concatenation 26 loci unphased	$2^{22} = 4194304$	$2^{9} = 512$
Concatenation 130 loci phased	$2^{21} = 2097152$	$2^{8} = 256$
Concatenation 130 loci unphased	$2^{20} = 1048576$	$2^{7} = 128$
*BEAST 26 loci	$2^{24} = 16777216$	$2^{11} = 2048$
StarBEAST2 26 loci	$2^{24} = 16777216$	$2^{11} = 2048$
StarBEAST2 52 loci	$2^{23} = 8388608$	$2^{10} = 1024$
StarBEAST2 50 loci – *Crocidura*	$2^{24} = 16777216$	$2^{11} = 2048$
StarBEAST2 100 loci – *Crocidura*	$2^{23} = 8388608$	$2^{10} = 1024$

3 CoordinatedUniform proposal example

The CoordinatedUniform operator will pick one non-leaf, non-root species tree node uniformly at random to be moved, dubbed S. Given a species tree with the topology ((A,B),C),D there are two non-leaf, non-root nodes: the common ancestor of species A and B (AB) and the common ancestor of species A, B and C (ABC). We illustrate an example where the ABC node is chosen (Figure S13).

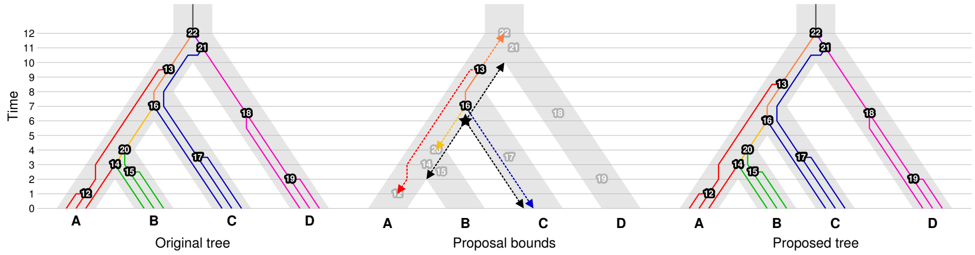

Figure S13: Illustration of the CoordinatedUniform move. An example gene tree is embedded within a four-taxon asymmetric species tree. Internal gene tree nodes are numbered, species leaves lettered. The ABC node is represented by a star in the middle step. Dashed lines are connected component and ABC node child and parent branches.

The step after selecting a species tree node is to determine which internal gene tree nodes will also be moved. CoordinatedUniform moves every gene tree node s in every gene tree which meets all of the following requirements:

1. at least one descendant individual of s is also a descendant individual of the *left* child of S
2. at least one descendant individual of s is also a descendant individual of the *right* child of S
3. all descendent individuals of s are also descendent individuals of S

Descendent individuals of a gene tree node s are gene tree leaf nodes which have s as an ancestor. Descendent individuals of a species tree node S are gene tree leaf nodes belonging to any extant species which has S as an ancestor. In the example, the left child of S is the AB node, and the right child of

S is the C node. The following internal nodes in the gene tree meet requirement (1) 12, 13, 14, 15, 16, 20, 22; requirement (2) 13, 16, 17, 21, 22; and requirement (3) 12, 13, 14, 15, 16, 17, 20.

The only nodes in the example meeting all three requirements are 13 and 16. These nodes are connected by a gene tree branch, making them a single connected component. The connected component has three child branches (dashed red, yellow and blue lines) and one parent branch (dashed orange line).

To determine the lower bound for the move, we must first identify the shortest branch out of all connected component child branches and species tree node S child branches. In the example the connected component child branches have lengths of 3, 7 and 8.5, and the species tree node child branches have lengths 4 and 6 (Figure S13). The lower bound is therefore the original species tree node height $t(S) - 3 = 6 - 3 = 3$.

To determine the upper bound we use the shortest branch out of all connected component parent branches and the species tree node S parent branch. The parent branch of the single connected component has a length of 2.5, and the parent branch of S has a length of 4. The upper bound is therefore $t(S) + 2.5 = 6 + 2.5 = 8.5$.

A new species tree node height $t'(S)$ between 3 and 8.5 is chosen uniformly at random. Within this range, if the connected component node heights are changed by $\eta = t'(S) - t(S)$, no topology changes are induced or required. In our example the new height is 5, so $\eta = 5 - 6 = -1$. After the height of the species tree node S and all connected component gene tree nodes are changed by $\eta = -1$ the proposal is complete (Figure S13).
